# The Involvement of Neutrophil Extracellular Traps in Disease Activity Associated With IgA Vasculitis

**DOI:** 10.3389/fimmu.2021.668974

**Published:** 2021-09-03

**Authors:** Xiu-Qi Chen, Li Tu, Jia-Sen Zou, Shi-Qun Zhu, Yan-Jun Zhao, Yuan-Han Qin

**Affiliations:** Department of Pediatrics, The First Affiliated Hospital, Guangxi Medical University, Nanning, China

**Keywords:** neutrophil extracellular traps, IgA vasculitis, children, IgAV nephritis, pathogenesis

## Abstract

**Objectives:**

This aim of this study was to determine whether neutrophil extracellular traps (NETs) are involved in the pathogenesis of IgA vasculitis (IgAV) and investigate whether the circulating NETs levels are associated with disease activity in children.

**Methods:**

We performed a case-control study and collected blood samples from 193 children with different stages of IgAV (61 were at the onset stage, 64 at the remission stage, 43 at the active stage, and 25 were undergoing drug withdrawal). A total of 192 healthy children were recruited as controls. Circulating cell free DNA (cf-DNA) was obtained from the plasma and quantified by using the Quant-iT PicoGreen DNA quantification kit. NETs-associated myeloperoxidase-DNA (MPO-DNA), citrullinated-histone H3 (cit-H3), neutrophil elastase (NE), and the deoxyribonuclease I (DNase I) concentrations were measured using enzyme-linked immunosorbent assays. The presence of NETs in the kidney and gastrointestinal tissues of onset and active IgAV patients was determined by multiple immunofluorescence staining in 15 IgAV nephritis patients and 9 IgAV patients without IgAV nephritis, respectively. NETs degradation potency of collected sera samples from IgAV patients were checked *in vitro*. Relationships between circulating levels of cf-DNA with MPO-DNA, NE, and DNase I and the patients were analyzed.

**Results:**

Circulating levels of cf-DNA in onset and active IgAV patients were significantly higher than those in remission and drug withdrawal patients as well as healthy controls. The results were similar for MPO-DNA and NE. The levels of circulating cf-DNA correlated significantly with MPO-DNA, NE and DNase I. A significantly decreased degradation of NETs from the onset and active IgAV patients was observed, but was normal in healthy controls. Furthermore, presence of NETs was also confirmed in all renal and gastrointestinal tissues obtained from the onset and active IgAV patients but not control samples.

**Conclusions:**

Our data showed that NETs were released into the circulation of IgAV patients and are involved in the disease activity. The circulating levels of NETs maybe used to assess disease severity in children with IgAV.

## Introduction

Immunoglobulin A vasculitis (IgAV) that is characterized by non-thrombocytopaenic purpura, gastrointestinal hemorrhage, and glomerulonephritis is the most common type of systemic vasculitis which occurs in childhood (1). IgAV, formerly called Henoch-Schönlein purpura, is a disease that causes IgA to collect in small blood vessels, which then become inflamed and leak blood. The incidence of IgAV is 20–70/100,000 per year in children (2). Almost 90% of cases occur between the ages of 2 and 10 years, and the peak incidence is from 4 to 7 years (3). IgAV is a self-limited disease and most patients have a benign prognosis. However, pediatricians should be aware of the possibility of early gastrointestinal morbidity as well as late-stage renal morbidity. In a small number of renal-associated IgAV patients who showed repeated symptoms, this disease was found to be the main cause of death.

So far, the etiology of IgAV have not been fully elucidated, and it is believed that the disease may be related to infection, vaccination, drugs, and inheritance. The pathogenesis of IgAV is still not very clear and may be linked to the abnormal dysregulated immune response to antigens mediated by IgA. It has been shown that galactose-deficient IgA1 can be recognized by anti-glycan antibodies in IgAV patients, which results in the formation of circulating immune complexes (ICs) that are subsequently deposited in the wall of small blood vessels leading to an inflammatory reaction and renal tissue injury (4).

Neutrophil extracellular traps (NETs), which are fibrous networks that protrude from the membranes of activated neutrophils, were first found by Brinkmann et al. in 2004 (5). These are found in a variety of conditions including infection, malignancy, atherosclerosis, and autoimmune diseases, and they contain cell free DNA (cf-DNA), myeloperoxidase (MPO), histone, neutrophil elastase (NE), and several other antibacterial proteins (6). NETs are involved in the development of autoimmunity by breaking down the self-tolerance of the immune system. They may also be potentially useful as markers in order to assess the development and prognosis of autoimmune diseases (7, 8). The study by Bergqvist and co-workers showed that NETs were higher in dermal tissues in the early phases of IC-mediated small vessel vasculitis in hypersensitivity vasculitis and IgAV (9). However, there are no previous studies to evaluate the effects of NETs in either the circulation or in renal and gastrointestinal tissues in IgAV. Therefore, the aim of this study was to explore whether NETs are involved in the pathogenesis of IgAV in children. In particular, the relationships between the levels of circulating cf-DNA were examined during the onset phases of IgAV.

## Materials and Methods

### Study Design and Participants

This study was approved by the ethics committee of the First Affiliated Hospital of Guangxi Medical University (NO. 2019 KY-E-137), and written informed consent was obtained from all the parents or guardians of the patients involved.

A case–control study was performed to evaluate levels of NETs in IgAV and whether circulating NETs could predict the activity of IgAV. This study included 193 cases IgAV in children and 192 healthy control children who were recruited from February 2019 to May 2020 in the First Affiliated Hospital of Guangxi Medical University. The IgAV patients were divided into four sub-groups: 61 patients were allocated to the onset group, 64 patients were in the remission group, 43 patients were allocated to the active group (which included some subjects undergoing relapse), and 25 patients were in the drug withdrawal group (withdrawal group).

The inclusion criteria were as follows: All the patients met the clinical diagnosis of IgAV after use of the EULAR/PRINTO/PRES criteria (10). Renal involvement (IgAV nephritis, IgAVN) was defined as the presence of hematuria (red cell count >5/mm^3^ at high magnification) and/or a proteinuria and/or an eGFR <60 ml/min in children (11). The onset group included the patient diagnosed with IgAV treatment without glucocorticoids and/or immuno-suppressor or treatment with glucocorticoids for less than 3 days for the first time, and the untreated active patients were those without glucocorticoids and/or immuno-suppressor treatment. The active/relapse group was defined as presence one or more of the following symptoms: new rash, arthralgia, abdominal pain, bloody stools, gastrointestinal bleeding, hematuria, and proteinuria, during the maintenance immunosuppressive therapy or after withdrawal of this treatment (i.e., an immunosuppressive therapy-free period). Remission was defined as patients without active/relapse of the disease as described above during their stable maintenance through immunosuppressive therapy periods. The drug withdrawal group was defined as “clinical cured” patients who were neither undergoing current drug treatment nor having regular follow-ups during their drug-free periods.

The exclusion criteria were the following: (1) absence of skin lesions, (2) thrombocytopenia (platelet <100,000/mm^3^), and (3) presence of any other immune and infection diseases such as respiratory and urinary tract infections, sepsis and systemic inflammatory response syndrome.

### Sample Collection and Analysis

Peripheral venous blood samples from the IgAV patients and healthy control were collected into tubes containing ethylene diamine-tetra-acetic acid (EDTA) anticoagulating agent and centrifuged at 1000g for 10 min at 4°C. After centrifugation, the supernatant plasma was separated and then stored at -80°C until use. Fifteen IgAVN patients’ renal tissue samples and 9 IgAV patients’ (with gastrointestinal involvement and without IgAVN) gastrointestinal tissue samples were formalin-fixed. These were then paraffin-embedded into blocks, and these were used to evaluate the status of NETs. All the patients who were subjected to renal puncture and gastrointestinal endoscopy had to undergo these procedures in order to verify their medical evaluation before the initiation of treatment. The biopsies pertinent to the study were taken during these procedures after consent of the parties involved.

Controls renal biopsies were taken from the normal tissues adjacent to the presence of tumors of 5 patients who underwent surgery for nephrocytoma. Controls gastrointestinal biopsies were taken from five samples of that anatomical area of healthy children with normal endoscopy and histology who were undergoing routine treatment for other reasons.

### Quantification of Circulating NETs Level and DNase I

It is known that NETs are composed of cf-DNA, MPO, NE, and histones, including citrullinated histone H3 (cit-H3). NETs are also the major source of circulating cf-DNA. To identify NETs, we quantified the cf-DNA, cit-H3, MPO-DNA, and NE in plasma samples as was done in a previous study (12). The cf-DNA was quantified by fluorescent assays using ds-DNA Quant-iT PicoGreen quantification kit (Invitrogen). The tests were performed according to the manufacturer’s instructions. Briefly, a DNA standard curve of DNA was constructed by using a range of dilutions from 1 to 1000 ng/ml and these samples were incubated for 5 min with Quant-iT PicoGreen reagent at room temperature. The plasma samples to be tested were diluted to 1:5, and the fluorescence signals obtained were measured using a microplate fluorescence reader (Thermo Fisher Spectrophotometer 1510) with filter settings set at wavelengths of 480 and 520nm for excitation and emission, respectively.

Plasma levels of MPO-DNA, NE, and DNase I were determined by enzyme-linked immunosorbent assays (ELISAs). The human MPO-DNA Quantikine ELISA Kit, human PMN elastase ELISA kit, and human deoxyribonuclease I, ELISA Kit used were purchased from Cusabio, Wuhan, 430071. Briefly, after preparing the standards and diluted samples (1:5), the plates were covered with an adhesive strip and incubated for 60 min at 37°C. Horseradish peroxidase-conjugated antibody specific for MPO-DNA (100 µl) was added and incubated for 60 min at 37°C. After washing five times with 200 μl of wash buffer, 90 µl of 3,3’,5,5’-tetramethylbenzidene substrate was added and incubated for a further 20 min at 37°C in dark. The O.D. absorbance at 540 nm was read in a microplate reader (Thermo Fisher spectrophotometer 3020) within 20 min after adding 50 µl of stop solution. In order to measure NE and DNase I, the methods were used in accordance with the manufacturer’s instructions.

### Isolation of Neutrophils and Degradation of NETs *In Vitro*

Neutrophils were isolated from healthy children which were then degraded to generate NETs *in vitro* according to a previously published method (13, 14). Neutrophils were freshly isolated from healthy children and seeded into a 96-well plate. Phorbol-12-myristate-13-acetate (20 nM) (Sigma) was added to the plate and incubated for 4 h at 37°C in an atmosphere of 5% CO_2_ so as to generate NETs. The sera from patients and control subjects were diluted in 10 mM Tris-HCl, pH 7.5, 50 mM NaCl, 10 mM MgCl_2_, and 2 mM CaCl_2_ and added to NETs, and these were incubated at 37°C for 60 min. Aliquots of the solution were then transferred to PBS to form a final concentration of 2 mM EDTA order to stop further degradation. The DNA concentration was quantified using Quant-iT PicoGreen quantification kit (Invitrogen) in accordance with the manufacturer’s instructions. A sample of pooled normal healthy children’s serum samples was used as an internal control. All samples were compared to the mean of the internal control, and they were measured in duplicate. The amount of degradation calculated for the positive and negative internal controls was set to 100 and 0%, respectively. The amount of degradation of NETs observed in serum samples was expressed as a percentage.

### Histology, Immunohistochemistry and Immunofluorescence Staining of NETs in Kidney and Gastrointestinal Tissues

Tissue samples for histology, immunohistochemistry, and immunofluorescence were taken from formalin-fixed, paraffin-embedded blocks. For general morphology and immuno-histochemical analysis, 5–7 μm thick sections were stained with hematoxylin and eosin (H&E) and Masson or hexamine silver stains. For immunofluorescence analysis studies, paraffin sections were de-paraffinized in xylene and dehydrated in graded ethanol solutions and then incubated for 15 min at 60–70°C in EDTA antigen-retrieval buffer (pH8.0). Slides were cooled to room temperature, and then rinsed in PBS (pH 7.4) three times. The endogenous peroxidase was blocked with 3% hydrogen peroxide solution and the sections were incubated at room temperature in the dark for 25 min. Samples were then washed with PBS three times and then blocked with 2% bovine serum albumin solution and normal goat serum for 30 min at room temperature. Samples were then incubated with primary antibodies dissolved in PBS and incubated at 4°C overnight. The following primary antibodies were used: rabbit anti-human NE (1:3000; Abcam, Cambridge, UK), rabbit anti-human MPO (1:1000; Abcam), and rabbit anti-human citH3 (1:100; Abcam). Slides were washed three times with PBS and incubated with CY3-TSA (Servicebio, Technology Co. Ltd., Wuhan, China; diluted 1:2000), Cy5 AffiniPure Goat Anti-Rabbit IgG (1:400, Servicebio), and FITC-TSA (1:1000, Servicebio) in PBS and incubated at 4°C for 50 min. Coverslips were mounted on glass slides using prolonged gold anti-fade reagent with DAPI (Servicebio) for 10 min to counterstain the DNA. Slides were washed a further three times for 5 min each time with PBS and covered with a water soluble fluorescence mounting medium (Servicebio). Images were acquired using 3 DHISTECH Digital slice scanning system (Pannoramic, 3DHISTECH Ltd., Hungary). Image analysis was performed by using the Image J software version 1.51a (NIH, Bethesda, MD). The percentage area occupied by NETs in the tissue was calculated as follows: area of NETs as determined by cit-H3 that co-localized with MPO/NE within the tissue/per high magnification 40× field. When multiple lesions were present in a specimen, the mean value were calculated.

### Statistical Analysis

Statistical analysis was performed with the Statistical Package for the Social Sciences software, release 16.0 for Windows (SPSS 16.0, USA). The Shapiro-Wilk test was used to determine the normality of the data which were expressed as the means ± standard deviations. Categorical variables were expressed as percentages. Differences of categorical data were assessed using the chi-square test. Differences of quantitative data were performed using Student’s t test, or one-way ANOVA between more than two groups and followed by a *post-hoc* Dunnett’s T3 test and the mean difference (MD) and standard error of the mean are given (SEM). Pearson’s or Spearman’s tests were used for correlation analysis as appropriate. A P value of <0.05 was considered as statistically significant.

## Results

### Clinical Characteristics of the Participants

Among the IgAV patients, there were 108 males and 85 females. The mean age of the patients at disease onset was 5.66 + 2.82 years. Among of the healthy controls, which consisted of 98 males and 93 females, the average age was 4.3 ± 1.2 years. There was no difference in gender and age between the IgAV patients and healthy control group. The baseline clinical characteristics of the patients in the different subgroups are shown in **Table 1**.

**Table 1:** Clinical characteristics of the participants in this study.

	Onset group	Remission group	Active group	Withdrawal group
No. of patients	61	64	43	25
Male (N %)	37 (60.1)	34 (53.1)	22 (51.1)	15 (60)
Age (y)	5.3 ± 1.8	7.6 ± 2.7	7.1 ± 3.2	8.6 ± 2.7
Disease duration (m)	1.1 ± 2.3	9.1 ± 10.9	11.15 ± 8.9	11.8 ± 11.7
Current treatment				post-treatment
Without glucocorticoids	49 (80.3)	17 (26.6)	8 (18.6)	7 (28)
Glucocorticoids	12(19.7)	31 (48.4)	26 (60.5)	15 (60)
Glucocorticoids + an immuno-suppressor	0	16 (25)	9 (20.9)	3 (12)
Skin involvement (N %)	61 (100)	64 (100)	43 (100)	25 (100)
Arthralgia (N %)	19 (31.1)	13 (20.3)	5 (11.6)	5 (20)
Abdominal involvement (N %)	45 (73.8)	27 (42.1)	26 (60.5)	18 (72)
Renal involvement (N %)	26 (42.6)	56 (87.5)	32 (74.4)	21 (84)
C-reactive protein (mg/L)	17.5 ± 11.9	9.7 ± 5.6	16.5 ± 9.6	9.2 ± 2.9
Erythrocyte sedimentation rate (mm/h)	22.1 ± 9.4	13.7 ± 5.6	25.5 ± 14.2	14.2 ± 2.9

### Circulating cf-DNA Levels Were Elevated During the Onset and Active IgAV Phases

One-way ANOVA was used to analyze differences between the IgAV subgroups and the control group. The results indicated that circulating cf-DNA levels were significantly different among the IgAV subgroups and control group (F = 122.29, P = 0.000). The *post-hoc* multiple comparison tests using Dunnett T3 test were conducted to evaluate the significance of the differences in any two groups. The *post-hoc* tests showed higher circulating cf-DNA levels in the onset group (327.52 ± 64.31 ng/ml) compared with the withdrawal group (208.8 ± 62.4 ng/ml). The MD and SEM between the two groups was 129 ± 12.8 ng/ml, P = 0.000. The results were similar when comparing the onset group (327.52 ± 64.31 ng/ml) with the remission group (200.47 ± 54.42 ng/ml, the MD and SEM were 127.1 ± 10.68 ng/ml, P = 0.000) or control group (187.91 ± 36.38 ng/ml, the MD and SEM were 139.6 ± 8.6 ng/ml, P = 0.000). No difference was found between the onset group and active group (315.15 ± 73.43 ng/ml), and the MD and SEM were 12.4 ± 13.9 ng/ml, P = 0.990.

**Figure 1:**
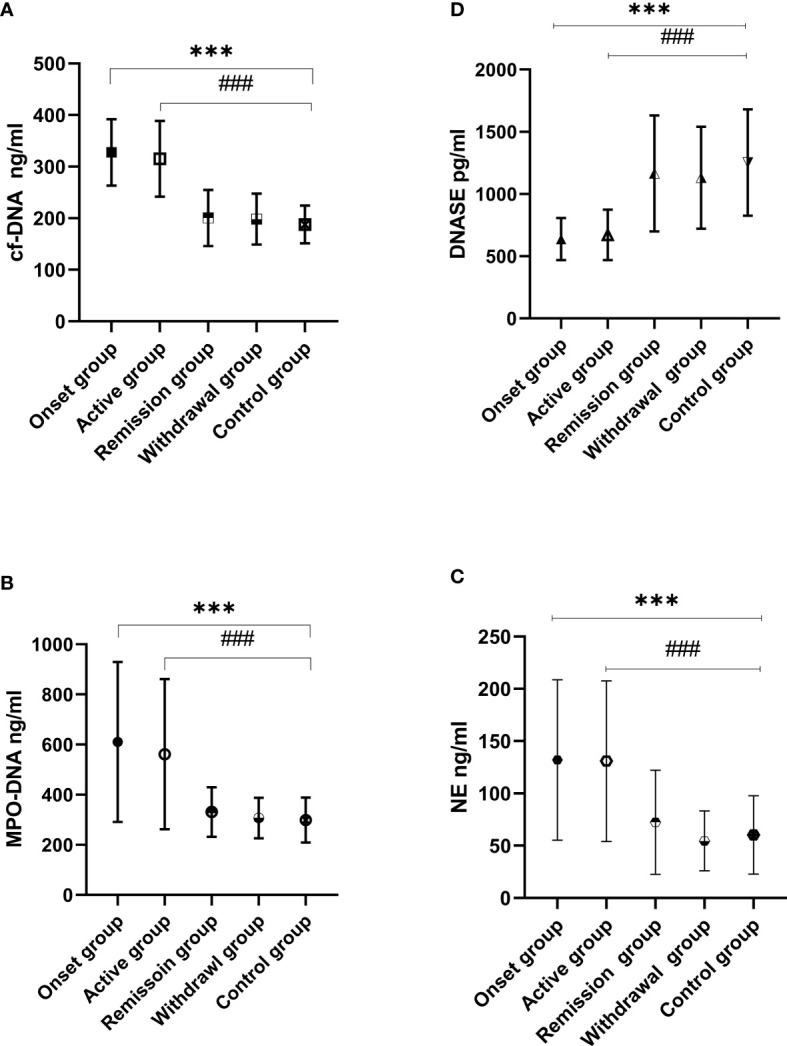
Circulating cf-DNA, MPO-DNA, NE, and DNase I levels were assessed in IgAV and control groups. cf-DNA **(A)**, MPO-DNA **(B)**, and NE **(C)** were elevated in the IgAV onset and active groups when compared to the remission, withdrawal, and control groups (^###^P < 0.001, ***P < 0.001). There were no differences observed between the remission, withdrawal, and control groups (P > 0.05 in all cases). The results were the opposite in DNase I levels **(D)** which were lower in the onset and active groups when compared with those in the withdrawal, remission, and control groups (^###^P < 0.001, ***P < 0.001).

The data also showed higher circulating cf-DNA levels in the active group compared with the withdrawal group (the MD and SEM were 116.8 ± 14.9 ng/ml, P = 0.000) or control group (the MD and SEM were 127.2 ± 11.5 ng/ml, P = 0.000). The results were similar when comparing the active group with remission group (the MD and SEM were 114.7 ± 13.1 ng/ml, P = 0.000). Analysis of the circulating cf-DNA levels of the withdrawal group and the remission group showed no significant difference (the MD and SEM were 2.1 ± 11.9 ng/ml, P = 1.0). Neither were there any differences observed between the two groups and the control group (**Figure 1**).

### MPO-DNA and NE Levels Were E levated in Onset and Active IgAV Patients

MPO-DNA and NE levels were significantly different among the IgAV patients’ subgroups and the control group when analyzed by one-way ANOVA. The *post-hoc* multiple comparison tests showed higher MPO-DNA and NE levels in the onset group (MPO-DNA and NE were 613.54 ± 318.7 and 134.83 ± 76.27 ng/ml, respectively) compared with the withdrawal group (MPO-DNA and NE were 306.19 ± 80.72 and 54.54 ± 28.67 ng/ml, respectively; the MD and SEM were 306.64 ± 43.88 and 80.3 ± 11.3 ng/ml, respectively, P = 0.000 in both cases), the remission group (MPO-DNA and NE were 330.89 ± 98.93 and 72.45 ± 49.78 ng/ml, respectively; the MD and SEM were 282.66 ± 42.64 and 62.4 ± 11.6 ng/ml, respectively, P = 0.000 in both cases), or the control group (MPO-DNA and NE were 298.96 ± 89.77 and 60.25 ± 37.49 ng/ml, respectively; the MD and SEM were 314.58 ± 41.31 ng/ml and 74.6 ± 10.1 ng/ml, respectively, P = 0.000 in both cases).

The results were similar when comparing patients of the active group (MPO-DNA and NE were 561.65 ± 299.57 and 148.78 ± 61.39 ng/ml, respectively) with the withdrawal group (the MD and SEM were 254.74 ± 45.12and 94.2 ± 10.9 ng/ml, respectively, P = 0.000 in both cases) and the remission group (the MD and SEM were 230.75 ± 35.38 and 76.3 ± 11.2 ng/ml, respectively, P = 0.000 in both cases). However, no difference was found when compared to the remission, withdrawal, and control groups (P > 0.05 in all cases; **Figure 1**).

**Figure 2:**
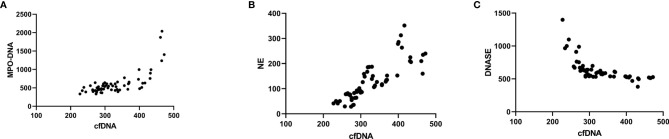
Correlation analysis of MPO-DNA, NE, and DNase with cf-DNA in isolated NETs. Correlation analysis showing that MPO-DNA **(A)** and NE **(B)** were highly correlated with circulating cf-DNA levels (MPO-DNA, r^2^ = 0.766, P < 0.001, NE, r^2^ = 0.687, P < 0.001). The opposite was found between cf-DNA and DNase levels **(C)** (r^2^ = 0.433, P < 0.001).

The results of correlation analysis showed that MPO-DNA and NE were highly correlated with circulating cf-DNA. The cf-DNA levels were positively correlated with MPO-DNA (r^2^ = 0.766, P < 0.001). The results were similar between the cf-DNA and NE levels (r^2^ = 0.687, P < 0.001; **Figure 2**).

### DNase I Levels Were in Declined in Onset and Active IgAV Patients

The DNase I levels were significantly decreased in the IgAV patients’ subgroups when compared with the control group. The *post-hoc* multiple comparison analysis of the IgAV patients showed lower DNase I levels in the onset (637.59 ± 169.17 pg/ml) and active groups (671.64 ± 202.01 pg/ml) when compared with those in the withdrawal (1130.85 ± 409.93 pg/ml) and remission groups (1165.71 ± 465.83 pg/ml) as well as in healthy subjects (1253.25 ± 427.03 pg/ml; P < 0.001 in all cases; **Figure 1**). The cf-DNA levels in NETs were negative correlated DNase I (r^2^ = 0.433, P < 0.001; **Figure 2**).

### The Ability to Degrade NETs Was Decreased in IgAV Patients

**Figure 3:**
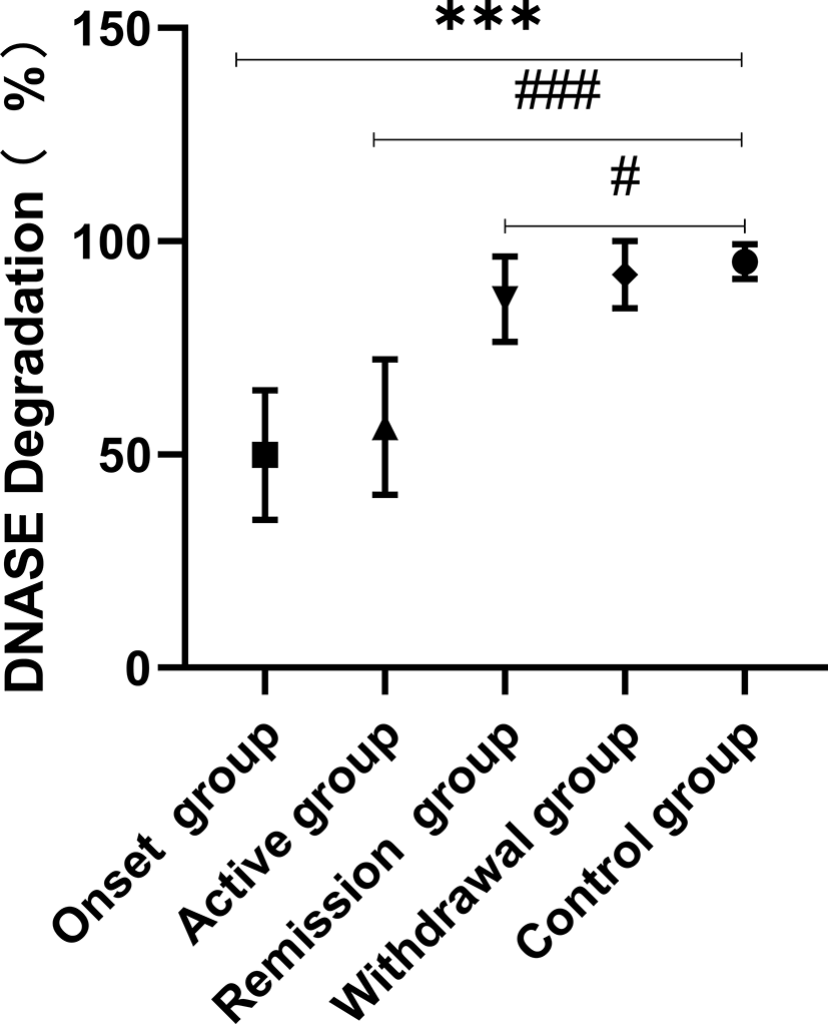
NETs were produced by PMA stimulation and degradation of NETs by the IgAV plasma were performed *in vitro*. Plasma from onset and active IgAV patients had decreased abilities to degrade NETs. The results showed significantly lower ratios of degradation of NETS in onset and active groups (^###^P < 0.001, ***P < 0.001). Although the remission group showed some improvement in of degradation ability, they did not return to normal levels when compared to the control group (^#^P = 0.035). The ability to degrade NETs was not significantly different between the withdrawal and normal groups (P > 0.05).

The ability of serum from IgAV patients to degrade NETs was decreased when compared with healthy control. Sera from the onset and active groups had decreased abilities to degrade NETs. Analysis of the onset (49.88 ± 15.21%) and active groups (58.05 ± 14.14%) showed significantly lower ratios of degradation of NETs compared with the control group (95.31 ± 4.07%), P < 0.001 in both cases. Although the remission group (86.48 ± 10.0%) showed improvement in degradation ability, the levels observed in these patients did not return to normal when compared to the healthy control group (P = 0.035). The ability to degrade NETs was not significantly different between the withdrawal and the control groups (P > 0.05; **Figure 3**).

### Immunohistochemistry and Presence of NETs in Kidney and Gastrointestinal Tissues of Onset and Active IgAV Patients

**Figure 4:**
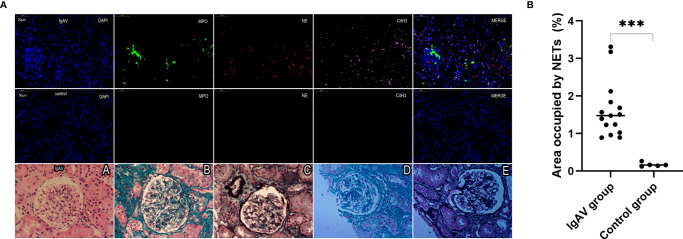
**(A)** Staining of renal tissue in order to visualize NETs and immuno-histochemical staining. Formalin-fixed, paraffin-embedded sections of renal tissue were subjected to immunofluorescence staining for MPO (green), NE (red), and cit-H3 (pink). DAPI was used for DNA staining and is shown in blue. NETs were observed in a pediatric IgAV patient but absent in control tissues. The sections were labeled for extracellular DNA, and cit-H3 was seen to co-localize with MPO/NE in the mesangial area of the glomeruli. Renal pathological types of IgAV were showed focal proliferative glomerulonephritis (A–E) and moderate mesangial hyperplasia glomerulonephritis (D, E) . The micrographs are from representative IgAV patients and control. **(B)** Area occupied by NETs in renal tissue, as determined by image analysis. ***P < 0.01.

NETs complexes were detected in renal specimens of 15 cases of IgAV at both the onset or active stages and in gastrointestinal specimens taken from 9 cases of IgAV with gastrointestinal involvement (i.e., without IgAVN) by multiple immunofluorescence analysis. The immunohistochemistry results from histological classification showed that 4 of the 15 IgAVN patients had minor degenerative nephritis, 9 expressed mesangial proliferation, and 2 had focal proliferative nephritis (**Figure 4A**). In all the IgAVN patients, IgA and C3 complex deposition were observed in the mesangial area of the glomeruli.

**Figure 5:**
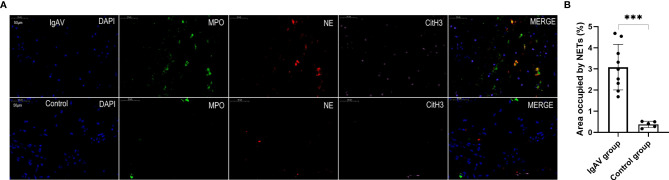
**(A)** Staining of intestinal tissue in order to visualize NETs. NETs were present in the villi mucosal and submucosal tissues of the duodenum of an IgAV patient but absent in healthy control tissue. The micrographs are from representative a IgAV patient and a healthy control. **(B)** Area occupied by NETs in intestinal tissue, as determined by image analysis. ***P < 0.01.

**Figure 6:**
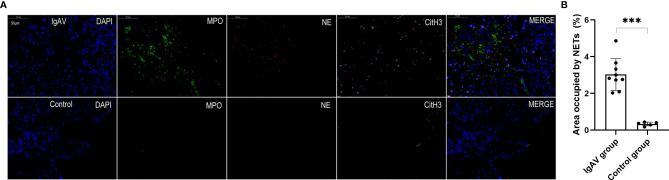
**(A)** Staining of gastric antrum tissue in order to visualize NETs. NETs were present in the mucosa of gastric antrum of an IgAV patient but absent in a healthy control tissue. The micrographs are from representative a IgAV patient and a healthy control. **(B)** Area occupied by NETs in gastric tissue, as determined by image analysis. ***P < 0.01.

NETs were also found in all the onset and active IgAV patients in both renal and gastrointestinal tissues. NETs were characterized by extracellular DNA and cit-H3 as localized with either MPO or NE in the involved mesangial areas of the glomeruli as well as gastrointestinal mucosal and submucosal tissues (**Figures 4A**, **5A**, and **6A**). These were the typical characteristics for NETs seen in IgAV patients. This was confirmed by the overlap with diffused DNA, and NETs deposition was evident in the onset and active IgAV patients. There was no evidence for NETs formation and deposition in control healthy children, who had normal renal and gastrointestinal histology (**Figures 4A**, **5A**, and **6A** controls). The area occupied by NETs within the lesion in all the tissue was significantly positive in quantitative analysis compared to the control (**Figures 4B**, **5B**, and **6B**, all P < 0.01).

## Discussion

This study evaluated NETs in both renal and gastrointestinal tissues and showed higher levels in onset and active IgAV patients when compared with control children. The study also showed higher circulating cf-DNA, MPO-DNA, and NE levels in NETs isolated from IgAV patients compared to controls. Moreover, we specifically evaluated the IgAV patients in subgroups and found that the levels of cf-DNA in onset and active IgAV patients were significantly elevated compared with those in remission or patients undergoing drug withdrawal as well as healthy subjects. This implies a high level of inflammation that is linked to disease activation or progression during onset and active IgAV. The accumulation of excessive NETs due to the imbalance between their formation and clearance can lead to chronic inflammation and tissue damage in autoimmune disorders. In this type of disease, the self-antigens exposed by NETs cause autoantibody production with increased risk of inflammation. All these processes are involved in the pathogenesis of autoimmune and inflammatory diseases (7). There are several studies which show that NETs are involved in autoimmune diseases such as anti-neutrophil cytoplasm antibodies (ANCA)-associated vasculitis, rheumatoid arthritis (RA), inflammatory bowel disease, and systemic lupus erythematosus (SLE) (15–18). Excessive NETs in IgAV patients are supported by the data presented here which confirms the view that altered NETs are linked to disease severity and that this phenomenon may be use as a marker of IgAV activity in children.

The presence of NETs formation in renal and gastrointestinal tissues was investigated by staining biopsy-obtained sections with multiple immunofluorescence markers using three DHISTECH Digital slice scanning systems. This scanning system can provide a higher resolution than the light microscopy and we found a greater number of NETs were present in renal glomerular tissues and the gastrointestinal mucosa of onset and active IgAV patients, but none in control group. The results suggested a role of NETs in pathogenesis of IgAV, but the effects of this process needs further research. IgA IC deposits are characteristic of IgAV and these can potently activate neutrophils, which results in the release of NETs. There is also evidence to show that NETs are involved in various IC-mediated small vessel vasculitis. The circulating amounts of NETs were strongly correlated with the severity of vessel inflammation (9). NETs formation was found in affected glomeruli and interstitial spaces of renal biopsies in ANCA‐associated vasculitis patients (19). An *in vitro* study also showed that NETs released *via* the Fcα receptor I (FcaRI) could be induced by IgA (20). IgA FcaRI was elevated in active IgAV patients (21). In spontaneous IgA nephropathy (IgAN), the proteinuria and leukocyte infiltration were severe due to marked inflammation activated by FcaRI. Moreover, IgA-IC induced NETosis and NETs released by activation of FcaRI resulted in TNF-α production. Thus, FcaRI activation promoted kidney damage by initiating a cytokine and chemokine cascade (22). Moreover, NETs could also induce autoimmunity in autoimmune diseases such as SLE (23). The impaired NETs degradation was associated with RA and lupus nephritis (24–27). In addition, the levels of circulating NETs were associated with periodontitis severity in patients with RA (28).

In the current study, we found that the ability to degrade NETs was decreased in onset and active IgAV patients. IgAV patients undergoing drug withdrawal had a normal ability to degrade NETs. DNase I levels were significantly decreased in the onset and active IgAV patients. The decreased degradation of NETs was correlated with presence of DNase I which was required to degrade NETs (14). Some studies have shown that impaired DNase I activity could lead to an immunological imbalance (29). Analysis of NETs showed weaker degradation of extracellular DNA in eosinophylic granulomatosis with polyangiitis associated with SLE patients. This phenomenon appears to be a characteristic of autoimmune disease (30, 31). DNase I was inhibited by complement over-activation as well as excessive deposition of the complement protein, C1q, which resulted in the inability to degrade NETs (13). The circulating DNA accumulation is probably due to a deficiency of the DNase I activity which can promote immune disorders and tissue damage (32). Elevated NETs is a characteristic of IgAV due to decreasing DNase activity and the ability to degrade NETs as a result of the disease.

NETs formation was found in vasculitides biopsied in patients before the onset of IC-mediated vasculitis (7). NETs may play a role in pathogenesis of IgAV, although the mechanism by which NETs induce tissue damage is unknown. It maybe that the formation and increase of NETs in onset and active IgAV patients indicate activation of neutrophil-releasing NETs by IgA IC. The IC deposition plays a central role in the pathogenesis of IgAV through complement activation, neutrophilic infiltration and the release of destructive enzymes which, in turn, lead to vessel damage. Immobilized ICs can induce the release of NETs from human neutrophils *in vitro* (33). Another NETosis pathways reported in lupus nephritis is that the circulating IC deposits in the glomerular basement membrane is accompanied by NETs accumulation resulting in tissue damage (34). This study found elevated NETs in the renal glomeruli of onset and active IgAV patients, and it is also known that IgA-IC deposition in the glomerular mesangial region is a characteristic of IgAV. The NETs were only elevated in active or onset IgAV patients but were normal in those in remission and undergoing drug withdrawal in this study. Taken together, these findings would suggest that NETs may be useful as a prognostic or diagnostic indicator for disease severity in IgAV patients. This could allow early assessment so as to not to stop medication of IgAV patients during the remission stage of the disease. In addition, assessments could allow more aggressive treatments to be targeted for the active IgAV patients with high NETs, particularly in relation to subjects with a poor renal prognosis. Some previous studies have focused on cfDNA as a predictor (35, 36), but Moss et al. study showed that cfDNA can be released during inflammatory disease from multiple cell types other than neutrophils (37). It is important to emphasize that NETs should act as a maker for disease prediction and not simply cfDNA.

However, this study also has some limitations. Firstly, this was a single center case-control study. The sample size is relatively small with respect to the subgroup analysis, and our data may be biased due to the small sample size of renal and gastrointestinal biopsies. Secondly, we did not obtain renal and gastrointestinal tissues from remission and withdrawal IgAV patients for the ethical reasons. However, this study provides the evidence for the presence of NETs in onset and active IgAV patients, particularly in those where renal and gastrointestinal mucosal biopsies were obtained.

In conclusion, this preliminary study shows that IgAV patients have increased NETs levels during the onset and active stages when compared with those patients in remission and withdrawal stages. This suggests a higher systemic inflammatory status in onset and active IgAV patients and a possible role of NETs in the maintenance of its inflammatory milieu. NETs were shown to be involved in disease activity in IgAV vasculitis. The circulating NETs levels maybe used as a potential indicator for assessing IgAV severity and may help towards managing the patients’ treatment regimen. This study lays a foundation for more studies to be performed in order to confirm the conclusions and to elucidate the precise role of NETs in the mechanism of IgAV.

## Data Availability Statement

The raw data supporting the conclusions of this article will be made available by the authors, without undue reservation.

## Ethics Statement

The study was approved by the ethics committee of the First Affiliated Hospital of Guangxi Medical University (no. 2019 KY-E-137). Written informed consent to participate in this study was provided by the participants’ legal guardian/next of kin.

## Author Contributions

XQC contributed to the study design, performed the experiments, and drafted the paper. LT, JSZ, SQZ, and YJZ contributed sample collection and performed some parts of the experiments. YHQ contributed to the conception and design of the study and revised the final paper. All authors contributed to the article and approved the submitted version.

## Funding

This study was supported by the Innovation Project of Guangxi Graduate Education (no. YCBZ2021047), the Research Basic Ability Enhancement Project for Young and Middle-aged Teachers in Guangxi Universities (no: 2021KY0096), and the Guangxi Medical High-level Backbone Talent “139” Plan.

## Conflict of Interest

The authors declare that the research was conducted in the absence of any commercial or financial relationships that could be construed as a potential conflict of interest.

## Publisher’s Note

All claims expressed in this article are solely those of the authors and do not necessarily represent those of their affiliated organizations, or those of the publisher, the editors and the reviewers. Any product that may be evaluated in this article, or claim that may be made by its manufacturer, is not guaranteed or endorsed by the publisher.

## References

[1] Jennette JC Falk RJ Bacon PA Basu N Cid MC Ferrario F. 2012 Revised International Chapel Hill Consensus Conference Nomenclature of Vasculitides. Arthritis Rheum (2013) 65(1):1–11. 10.1002/art.37715 23045170

[2] Schnabel A Hedrich CM. Childhood Vasculitis. Front Pediatr (2018) 6:421. 10.3389/fped.2018.00421 30687686 PMC6335362

[3] Leung AKC Barankin B Leong KF. Henoch-Schonlein Purpura in Children: An Updated Review. Curr Pediatr Rev (2020) 16(4):265–76. 10.2174/1573396316666200508104708 32384035

[4] Lau KK Suzuki H Novak J Wyatt RJ. Pathogenesis of Henoch-Schonlein Purpura Nephritis. Pediatr Nephrol (2010) 25(1):19–26. 10.1007/s00467-009-1230-x 19526254 PMC2778786

[5] Brinkmann V Reichard U Goosmann C Fauler B Uhlemann Y Weiss DS. Neutrophil Extracellular Traps Kill Bacteria. Science (2004) 303(5663):1532–5. 10.1126/science.1092385 15001782

[6] Lee KH Kronbichler A Park DD Park Y Moon H Kim H. Neutrophil Extracellular Traps (NETs) in Autoimmune Diseases: A Comprehensive Review. Autoimmun Rev (2017) 16(11):1160–73. 10.1016/j.autrev.2017.09.012 28899799

[7] Fousert E Toes R Desai J. Neutrophil Extracellular Traps (NETs) Take the Central Stage in Driving Autoimmune Responses. Cells (2020) 9(4):915–24. 10.3390/cells9040915 PMC7226846 32276504

[8] Mulay SR Anders HJ. Neutrophils and Neutrophil Extracellular Traps Regulate Immune Responses in Health and Disease. Cells (2020) 9(9):2130–3. 10.3390/cells9092130 PMC7565859 32962213

[9] Bergqvist C Safi R El Hasbani G Abbas O Kibbi A Nassar D. Neutrophil Extracellular Traps Are Present in Immune-Complex-Mediated Cutaneous Small Vessel Vasculitis and Correlate With the Production of Reactive Oxygen Species and the Severity of Vessel Damage. Acta Derm Venereol (2020) 100(17):adv00281. 10.2340/00015555-3363 31663600 PMC9274929

[10] Ozen S Pistorio A Iusan SM Bakkaloglu A Herlin T Brik R. EULAR/PRINTO/PRES Criteria for Henoch-Schonlein Purpura, Childhood Polyarteritis Nodosa, Childhood Wegener Granulomatosis and Childhood Takayasu Arteritis: Ankara 2008. Part II: Final Classification Criteria. Ann Rheum Dis (2010) 69(5):798–806. 10.1136/ard.2009.116657 20413568

[11] Schwartz GJ Munoz A Schneider MF Mak RH Kaskel F Warady BA. New Equations to Estimate GFR in Children With CKD. J Am Soc Nephrol (2009) 20(3):629–37. 10.1681/ASN.2008030287 PMC2653687 19158356

[12] Perdomo J Leung HHL Ahmadi Z Yan F Chong JJH Passam FH. Neutrophil Activation and NETosis are the Major Drivers of Thrombosis in Heparin-Induced Thrombocytopenia. Nat Commun (2019) 10(1):1322. 10.1038/s41467-019-09160-7 30899022 PMC6428879

[13] Leffler J Martin M Gullstrand B Tyden H Lood C Truedsson L. Neutrophil Extracellular Traps That are Not Degraded in Systemic Lupus Erythematosus Activate Complement Exacerbating the Disease. J Immunol (2012) 188(7):3522–31. 10.4049/jimmunol.1102404 22345666

[14] Hakkim A Fürnrohr BG Amann K Laube B Abed UA Brinkmann V. Impairment of Neutrophil Extracellular Trap Degradation Is Associated With Lupus Nephritis. Proc Natl Acad Sci USA (2010) 107(21):9813–8. 10.1073/pnas.0909927107 PMC2906830 20439745

[15] Moore S Juo HH Nielsen CT Tyden H Bengtsson AA Lood C. Role of Neutrophil Extracellular Traps Regarding Patients at Risk of Increased Disease Activity and Cardiovascular Comorbidity in Systemic Lupus Erythematosus. J Rheumatol (2020) 47(11):1652–60. 10.3899/jrheum.190875 PMC7520909 31839592

[16] de Bont CM Stokman MEM Faas P Thurlings RM Boelens WC Wright HL. Autoantibodies to Neutrophil Extracellular Traps Represent a Potential Serological Biomarker in Rheumatoid Arthritis. J Autoimmun (2020) 113:102484. 10.1016/j.jaut.2020.102484 32451286

[17] Abreu-Velez AM Smith JG Jr. Howard MS. Presence of Neutrophil Extracellular Traps and Antineutrophil Cytoplasmic Antibodies Associated With Vasculitides. N Am J Med Sci (2009) 1(6):309–13. PMC3364632 22666713

[18] Li T Wang C Liu Y Li B Zhang W Wang L. Neutrophil Extracellular Traps Induce Intestinal Damage and Thrombotic Tendency in Inflammatory Bowel Disease. J Crohns Colitis (2020) 14(2):240–53. 10.1093/ecco-jcc/jjz132 31325355

[19] Kessenbrock K Krumbholz M Schonermarck U Back W Gross WL Werb Z. Netting Neutrophils in Autoimmune Small-Vessel Vasculitis. Nat Med (2009) 15(6):623–5. 10.1038/nm.1959 PMC2760083 19448636

[20] Aleyd E van Hout MW Ganzevles SH Hoeben KA Everts V Bakema JE. IgA Enhances NETosis and Release of Neutrophil Extracellular Traps by Polymorphonuclear Cells *via* Fcalpha Receptor I. J Immunol (2014) 192(5):2374–83. 10.4049/jimmunol.1300261 24493821

[21] Moresco RN Speeckaert MM Zmonarski SC Krajewska M Komuda-Leszek E Perkowska-Ptasinska A. Urinary Myeloid IgA Fc Alpha Receptor (CD89) and Transglutaminase-2 as New Biomarkers for Active IgA Nephropathy and Henoch-Schonlein Purpura Nephritis. BBA Clin (2016) 5:79–84. 10.1016/j.bbacli.2016.02.002 27051593 PMC4802400

[22] Kanamaru Y Arcos-Fajardo M Moura IC Tsuge T Cohen H Essig M. Fc Alpha Receptor I Activation Induces Leukocyte Recruitment and Promotes Aggravation of Glomerulonephritis Through the FcR Gamma Adaptor. Eur J Immunol (2007) 37(4):1116–28. 10.1002/eji.200636826 17393381

[23] Bruschi M Bonanni A Petretto A Vaglio A Pratesi F Santucci L. Neutrophil Extracellular Traps (NETs) Profiles in Patients With Incident SLE and Lupus Nephritis. J Rheumatol (2020) 47(3):377–86. 10.3899/jrheum.181232 PMC6917988 31092713

[24] Bruschi M Petretto A Santucci L Vaglio A Pratesi F Migliorini P. Neutrophil Extracellular Traps Protein Composition Is Specific for Patients With Lupus Nephritis and Includes Methyl-Oxidized Alphaenolase (Methionine Sulfoxide 93). Sci Rep (2019) 9(1):7934. 10.1038/s41598-019-44379-w 31138830 PMC6538718

[25] Rother N Pieterse E Lubbers J Hilbrands L van der Vlag J. Acetylated Histones in Apoptotic Microparticles Drive the Formation of Neutrophil Extracellular Traps in Active Lupus Nephritis. Front Immunol (2017) 8:1136. 10.3389/fimmu.2017.01136 28959262 PMC5604071

[26] Sur Chowdhury C Giaglis S Walker UA Buser A Hahn S Hasler P. Enhanced Neutrophil Extracellular Trap Generation in Rheumatoid Arthritis: Analysis of Underlying Signal Transduction Pathways and Potential Diagnostic Utility. Arthritis Res Ther (2014) 16(3):R122. 10.1186/ar4579 24928093 PMC4229860

[27] Aleyd E Al M Tuk CW van der Laken CJ van Egmond M. IgA Complexes in Plasma and Synovial Fluid of Patients With Rheumatoid Arthritis Induce Neutrophil Extracellular Traps *via* FcalphaRI. J Immunol (2016) 197(12):4552–9. 10.4049/jimmunol.1502353 27913645

[28] Kaneko C Kobayashi T Ito S Sugita N Murasawa A Nakazono K. Circulating Levels of Carbamylated Protein and Neutrophil Extracellular Traps are Associated With Periodontitis Severity in Patients With Rheumatoid Arthritis: A Pilot Case-Control Study. PloS One (2018) 13(2):e0192365. 10.1371/journal.pone.0192365 29394286 PMC5796721

[29] Malickova K Duricova D Bortlik M Hruskova Z Svobodova B Machkova N. Impaired Deoxyribonuclease I Activity in Patients With Inflammatory Bowel Diseases. Autoimmune Dis (2011) 2011:945861. 10.4061/2011/945861 21687600 PMC3112520

[30] Pruchniak MP Ostafin M Wachowska M Jakubaszek M Kwiatkowska B Olesinska M. Neutrophil Extracellular Traps Generation and Degradation in Patients With Granulomatosis With Polyangiitis and Systemic Lupus Erythematosus. Autoimmunity (2019) 52(3):126–35. 10.1080/08916934.2019.1631812 31257985

[31] Masuda S Nonokawa M Futamata E Nishibata Y Iwasaki S Tsuji T. Formation and Disordered Degradation of Neutrophil Extracellular Traps in Necrotizing Lesions of Anti-Neutrophil Cytoplasmic Antibody-Associated Vasculitis. Am J Pathol (2019) 189(4):839–46. 10.1016/j.ajpath.2019.01.007 30677396

[32] Napirei M Wulf S Mannherz HG. Chromatin Breakdown During Necrosis by Serum DNase1 and the Plasminogen System. Arthritis Rheum (2004) 50(6):1873–83. 10.1002/art.20267 15188364

[33] Granger V Peyneau M Chollet-Martin S de Chaisemartin L. Neutrophil Extracellular Traps in Autoimmunity and Allergy: Immune Complexes at Work. Front Immunol (2019) 10:2824. 10.3389/fimmu.2019.02824 31849989 PMC6901596

[34] Nishi H Mayadas TN. Neutrophils in Lupus Nephritis. Curr Opin Rheumatol (2019) 31(2):193–200. 10.1097/BOR.0000000000000577 30540580

[35] Dyer MR Chen Q Haldeman S Yazdani H Hoffman R Loughran P. Deep Vein Thrombosis in Mice Is Regulated by Platelet HMGB1 Through Release of Neutrophil-Extracellular Traps and DNA. Sci Rep (2018) 8(1):2068. 10.1038/s41598-018-20479-x 29391442 PMC5794752

[36] Margraf S Logters T Reipen J Altrichter J Scholz M Windolf J. Neutrophil-Derived Circulating Free DNA (Cf-DNA/NETs): A Potential Prognostic Marker for Posttraumatic Development of Inflammatory Second Hit and Sepsis. Shock (2008) 30(4):352–8. 10.1097/SHK.0b013e31816a6bb1 18317404

[37] Moss J Magenheim J Neiman D Zemmour H Loyfer N Korach A. Comprehensive Human Cell-Type Methylation Atlas Reveals Origins of Circulating Cell-Free DNA in Health and Disease. Nat Commun (2018) 9(1):5068. 10.1038/s41467-018-07466-6 30498206 PMC6265251

